# Protective effect of low-dose risedronate against osteocyte apoptosis and bone loss in ovariectomized rats

**DOI:** 10.1371/journal.pone.0186012

**Published:** 2017-10-18

**Authors:** Tingjun Ye, Peng Cao, Jin Qi, Qi Zhou, D. Sudhaker Rao, Shijing Qiu

**Affiliations:** 1 Shanghai Key Laboratory for Prevention and Treatment of Bone and Joint Diseases with Integrated Chinese-Western Medicine, Shanghai Institute of Traumatology and Orthopedics, Ruijin Hospital, Shanghai Jiaotong University School of Medicine, Shanghai, China; 2 Department of Orthopedics, Ruijin Hospital, Shanghai Jiaotong University School of Medicine, Shanghai, China; 3 Bone and Mineral Research Laboratory, Henry Ford Hospital, Detroit, Michigan, United States of America; INSERM, FRANCE

## Abstract

Osteocyte apoptosis is the first reaction to estrogen depletion, thereby stimulating osteoclastic bone resorption resulting in bone loss. We investigated the effects of two different risedronate (RIS) doses (high and low) on osteocyte apoptosis, osteoclast activity and bone loss in ovariectomized rats. Forty rats with ovariectomy (OVX) and sham ovariectomy (SHAM) were divided into 4 groups: 1) SHAM rats treated with saline (SHAM); 2) OVX rats treated with saline (OVX); 3) OVX rats treated with low-dose RIS (OVX-LR, 0.08 μg/kg/day); 4) OVX rats treated with high-dose RIS (OVX-HR, 0.8 μg/kg/day). All animals were sacrificed 90 days after surgery for the examinations of osteocyte apoptosis by caspase-3 staining, osteoclast activity by TRAP staining and bone volume by micro-CT scanning in lumbar vertebral cancellous bone. Both low and high dose RIS significantly reduced caspase-3 positive osteocytes, empty lacunae and TRAP positive osteoclasts in OVX rats. Although the difference in caspase-3 positive osteocytes was not significant between the OVX-LR and OVX-HR groups, numerically these cells were significantly more prevalent in OVX-HR (not OVX-LR) group than in SHAM group. TRAP positive osteoclasts were significantly higher in OVX-LR group than in SHAM or OVX-HR group. There was no significant difference in bone volume among the OVX-LR, OVX-HR and SHAM groups, but lower in OVX group alone. However, significant increase in trabecular thickness only occurred in OVX-LR group. We conclude that both low and high dose RIS significantly inhibit osteocyte apoptosis and osteoclast activity in OVX rats, but the low-dose RIS has weaker effect on osteoclast activity. However, low-dose RIS preserves cancellous bone mass and microarchitecture as well as high-dose RIS after estrogen depletion.

## Introduction

Estrogen depletion after menopause or ovariectomy (OVX) stimulates osteoclastic bone resorption, resulting in uncoupled bone remodeling in which the bone formed by osteoblasts is unable to compensate for the amount resorbed by osteoclasts [1, 2]. It has now become increasingly clear that the first reaction to estrogen depletion is osteocyte death by apoptosis [3], which may cause bone loss and impair bone quality via two different pathways. First, apoptosing osteocytes signal the neighboring viable osteocytes to synthesize receptor activator of NFkB ligand (RANKL), an osteoclastogenic cytokine that initiates bone resorption [4]. Excessive bone resorption is a major factor contributing to bone loss. Second, since apoptotic osteocytes are distributed extensively in the skeleton after estrogen depletion, removal of such a large number of apoptotic osteocytes by a single bone remodeling cycle is incomplete, especially in deep-seated interstitial bone that is less accessible to osteoclasts [5]. Consequently, the remaining apoptotic osteocytes would fragment into apoptotic bodies and undergo secondary necrosis, after which the cellular material is dispersed leaving empty lacunae [6, 7]. More commonly, osteocyte apoptotic bodies become mineralized and coalesce to completely fill the lacunar and canalicular spaces, resulting in hypermineralized acellular bone area referred to as micropetrosis [8, 9]. Since accumulation of empty lacunae and micropetrosis would severely compromise bone material properties [5, 9, 10], inhibition of osteocyte apoptosis may be beneficial to maintain bone mass and improve bone quality.

Bisphosphonates (BPs) have revolutionized the management of osteoporosis, especially for the prevention and treatment of vertebral and hip fractures [11, 12]. In the last 20 years, it is recognized that BPs have dual effects on bone. The first is to stop bone loss by reducing the number of osteoclasts due to preventing osteoclastogenesis and promoting osteoclast apoptosis [13, 14], and the second is to inhibit osteocyte and osteoblast apoptosis in various pathological conditions [15–17]. *In vitro* studies suggest that such dual effects of BPs are concentration dependent [18]. The anti-apoptotic effect on osteocytes is seen at much lower doses than that required for inhibiting osteoclast activity [16, 18]. However, such dose-dependent effects are not so obvious in *in vivo* studies [17]. Plotkin et al [18] reported that the dose of alendronate with antiresorptive effect can also inhibit osteocyte and osteoblast apoptosis in mice treated with glucocorticoids.

The low-dose BPs used *in vivo* is defined as ≥10-fold lower than the high-dose associated with an optimal anti-resorptive activity [16]. Qiu et al [17] reported that low-dose risedronate (RIS) significantly inhibit osteocyte apoptosis in rats by 15 days after ovariectomy. Although low-dose BPs may have an effect against osteocyte apoptosis [16–18], it remains unclear if low-dose BPs can reduce bone resorption and preserve bone mass after estrogen depletion. The purposes of this study were to determine the low *versus* high dose (as defined) effects of RIS on osteocyte apoptosis, osteoclast activity and bone loss in rats after OVX.

## Materials and methods

### Experimental design

This research was approved by the Institutional Animal Care and Use Committee (IACUC) of Ruijin Hospital. Forty 6 months old female Sprague-Dawley rats were purchased from Shanghai Slack Laboratory Animals Ltd (Shanghai, China) and housed in a room at 22°C and 60% humidity with a 12-hour light/dark cycle. The rats underwent either ovariectomy (OVX; n = 30) or sham operation (SHAM; n = 10) under pentobarbital (35 mg/kg, ip) anesthesia. Bilateral ovaries were removed for OVX rats. For SHAM rats, ovaries were exteriorized and then replaced in the abdominal cavity. The rats were divided into 4 groups of 10 rats each after surgery: 1) SHAM rats treated with saline as a vehicle; 2) OVX rats treated with saline as a vehicle; 3) OVX rats treated with low-dose RIS (OVX-LR); 4) OVX rats treated with high-dose RIS (OVX-HR). Low-dose RIS (0.08 μg/kg/day) was 1 order of magnitude lower than the high dose (0.8 μg/kg/day). Subcutaneous injections of saline and RIS were started 3 days before surgery and then continued every 3 days until the animals were sacrificed.

All animals were euthanized at 90 days after surgery. Euthanasia was performed by peritoneal injection of overdose Pentobarbital (100 mg/kg). The 1^st^ and 3^rd^ lumbar vertebrae (L1 and L3) were removed from each rat. L1 was used for micro-CT examination and L3 was fixed in 4% paraformaldehyte for 24 hours, decalcified in 10% ethylene-diamine tetraacetic acid disodium (EDTA-2Na) for 2–3 weeks, dehydrated with graded ethanol, embedded in paraffin and longitudinally sectioned into 5 μm for immunohistochemical and tartrate-resistant acid phosphatase (TRAP) examinations.

### Immunohistochemistry

Caspase-3 assay (Abcam, Cambridge, MA, USA) was used for the detection of apoptotic osteocytes. After deparaffinizing and rehydrating, the sections were placed in 3% hydrogen peroxide/methanol for 10 minutes to quench endogenous peroxidase. Heat induced epitope retrieval was performed by microwave oven heating in unmasking solution (0.01M citrate buffer, pH 6.0) for 20 minutes. The sections were immersed in diluted normal serum for 1 hour and incubated with primary antibody (rabbit antibody that recognizes cleaved caspase-3, 1:100 dilution), or as a negative control incubated with normal rabbit IgG, for 24 hours at 4°C. After that, the sections were incubated in a goat anti-rabbit biotinylated secondary antibody (1:100 dilution) for 1 hour. Immunoreactivity was visualized by immersion in a solution of 0.05% DAB and 0.01% H_2_O_2_ that generates a brown color. Sections were counterstained with 1% methyl green.

**Fig 1:**
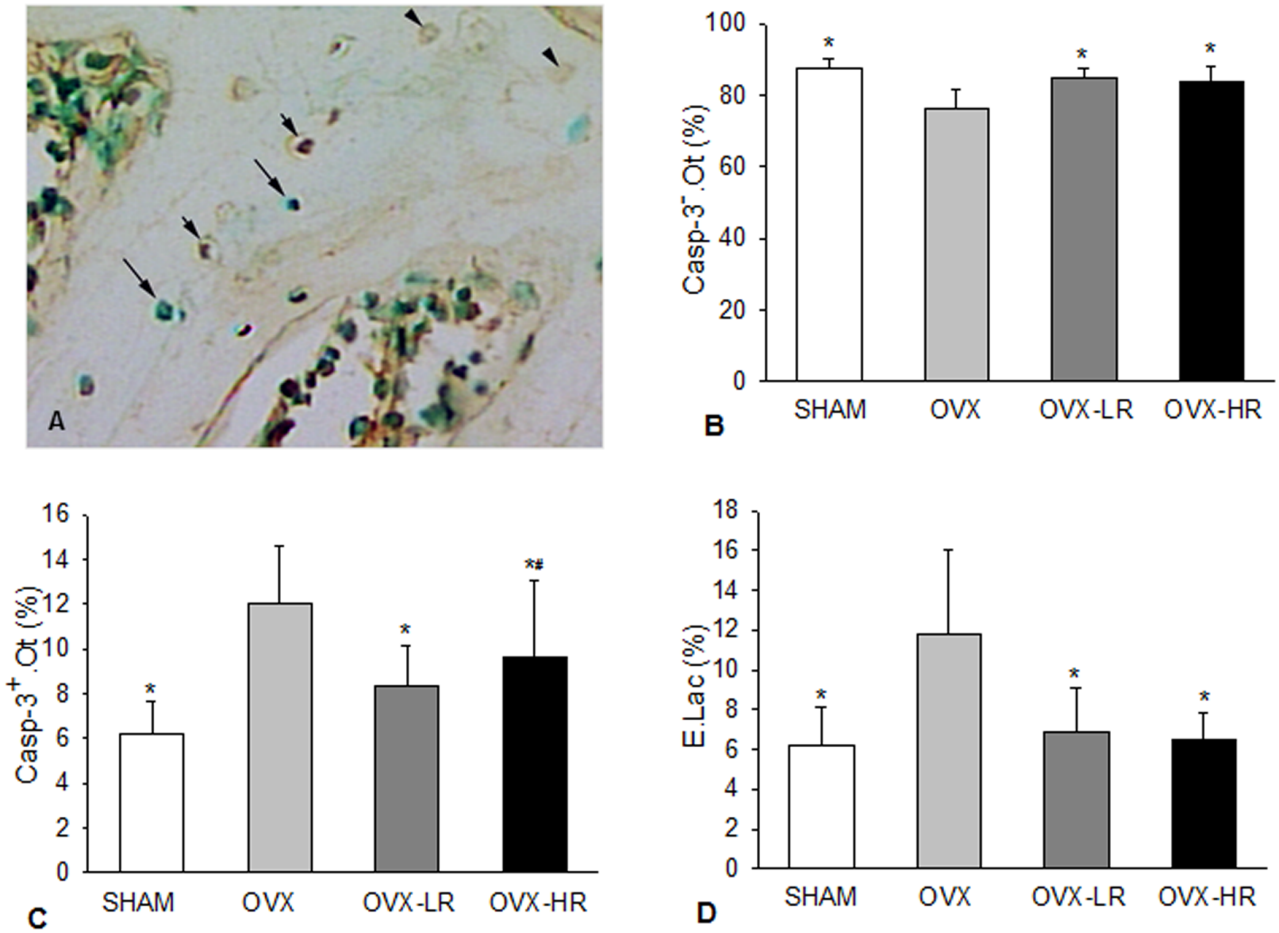
Both low and high doses of risedronate prevent osteocyte apoptosis resulting from estrogen depletion. A) Normal osteocytes (Casp^-^.Ot, long arrows), apoptotic osteocytes (Casp^+^.Ot, short arrows) and empty lacunae (E.Lac, arrow heads) shown in caspase-3 and methyl green stained section; B-D) Comparison of Casp^-^.Ot (*B*), Casp^+^.Ot (*C*) and E.Lac (*D*), respectively, among SHAM, OVX, OVX-LR and OVX-HR groups. *p < 0.05 *versus* OVX group; ^#^p < 0.05 *versus* SHAM group. Numerical data are presented in supporting information (S1 Table).

The vertebral sections were examined using a bright-field light microscope (20X objective) equipped with a Bioquant Image Analysis System (R&M Biometrics Inc., Nashville, TN, USA). Six areas were selected in different unbroken trabeculae for the measurements of caspase-3 positive osteocytes (Casp-3^+^.Ot), caspase-3 negative osteocytes (Casp-3^-^.Ot) as well as empty lacunae (E.Lac)(Fig 1A). The percentages of Casp-3^+^.Ot, Casp-3^-^.Ot and E.Lac were calculated in each area. Casp-3^+^.Ot and Casp-3^-^.Ot were regarded as apoptotic and non-apoptotic osteocytes, respectively.

### Tartrate-resistant acidic phosphatase (TRAP) staining

**Fig 2:**
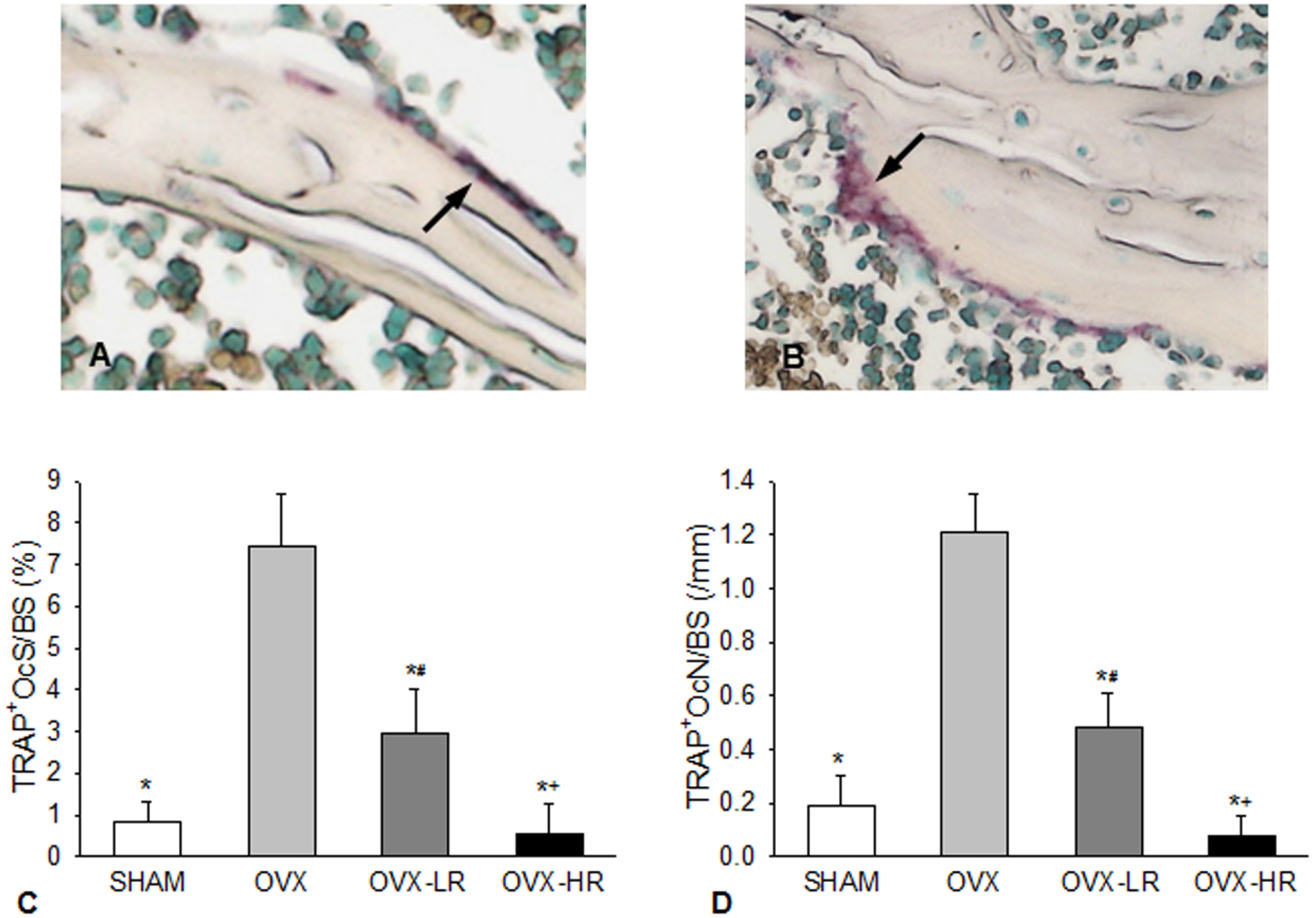
Both low and high doses of risedronate inhibit osteoclast activity resulting from estrogen depletion, but the effect is positively associated with the drug doses. A) TRAP-positive mononuclear cells on the bone surface (arrow); B) TRAP-positive multinuclear osteoclast on the bone surface (arrow); C&D) Comparison of TRAP positive osteoclast surface (TRAP^+^OcS/BS)(*C*) and TRAP positive osteoclast number (TRAP^+^Oc.N/BS)(*D*), respectively, among SHAM, OVX, OVX-LR and OVX-HR groups. *p < 0.05 *versus* OVX group; ^#^p < 0.05 *versus* SHAM group; ^+^p < 0.05 *versus* OVX-LR group. Numerical data are presented in supporting information (S2 Table).

TRAP staining was performed using a TRAP staining kit (Sigma, St. Louis, MO, USA) at room temperature. Slides were counterstained with fast green. The sections were examined using a bright-field light microscope (10X objective) equipped with a Bioquant Image Analysis System. Ten areas were selected on the surface of unbroken cancellous bone for the measurement of TRAP positive osteoclasts. In addition to multinuclear osteoclasts, TRAP-positive "mononuclear" cells or cells without a nucleus were also counted as osteoclasts (Fig 2A & 2B) as suggested by Ballanti et al [19].

1. TRAP-positive osteoclast surface (TRAP^+^OcS/BS; %): percentage of the trabecular surface in contact with TRAP-positive osteoclastic cells.
2. TRAP-positive osteoclast number (TRAP^+^OcN/BS; #/mm): number of TRAP-positive osteoclastic cells per millimeter of the trabecular surface.

### Micro-CT

A micro-CT system (μCT 80, Scanco Medical, Zurich, Switzerland) was used to scan the cancellous and cortical bone in L1 vertebral body with the following settings: tube voltage, 70 kV; tube current 0.1 mA and voxel size 15 μm. The manufacturer's software package was used for image processing and data evaluation (version 4.04). Each vertebra was scanned from cranial to caudal end at a slice thickness of 15 μm. Images of trabecular and cortical bone were segmented at 220 grayscale threshold value. We analyzed the middle portion (height, 2 mm) of the lumbar vertebral cylinder. Three-dimensional analysis was conducted using scanned slice data. Cortical and trabecular bones were separated manually using an in-house software.

Cancellous bone structure was determined by the parameters: cancellous bone volume fraction (Cn.BV/TV), trabecular thickness (Tb.Th), trabecular number (Tb.N) and trabecular separation (Tb.Sp). Cortical bone structure was determined by the parameters: cortical bone thickness (Ct.Th) and cortical bone volume fraction (Ct.BV/TV).

### Statistical analyses

One-way ANOVA with Tukey post-hoc test was used to compare the differences in each variable among the 4 groups as defined in the section of experimental design. For the non-normally distributed data, Kruskal–Wallis with Dunn's post hoc test was used for the comparisons. Pearson correlation analysis was used to determine the relationship between osteoclast and cancellous bone structure related variables. Results were expressed as mean ± SD for each group. A p-value <0.05 was considered significant. All analyses were performed using Sigma Plot 12.5 software program.

## Results

### Osteocyte apoptosis

Fig 1B–1D summarize the results for apoptotic osteocytes (Casp-3^+^.Ot), non-apoptotic osteocytes (Casp-3^-^.Ot) and empty lacunae (E.Lac) in groups of SHAM, OVX, OVX-LR and OVX-HR. There was significant difference in each variable among the 4 groups (p < 0.001 for all variables).

Apoptotic osteocytes and empty lacunae were significantly increased in the lumbar vertebral cancellous bone in OVX rats. Compared to SHAM rats, OVX caused a 93% increase in Casp-3^+^.Ot (Fig 1C) and a 90% increase in E.Lac (Fig 1D), resulting in a significant decrease in Casp-3^-^.Ot (Fig 1B).

Both low- and high-dose RIS significantly decreased osteocyte apoptosis resulting from OVX. Casp-3^+^.Ot (Fig 1C) and E.Lac (Fig 1D) were significantly lower in OVX-LR and OVX-HR groups than in OVX group. However, there were no significant differences in Casp^-^.Ot, Casp^+^.Ot and E.Lac between OVX-LR and OVX-HR groups (Fig 1B–1D). Post-hoc analysis showed that OVX-HR group, rather than OVX-LR, had significantly more Casp-3^+^.Ot as compared with SHAM group (Fig 1C).

### Osteoclast activity

Fig 2C & 2D summarize the results for TRAP^+^OcS/BS and TRAP^+^OcN/BS in the 4 groups. There was significant difference in each variable among the 4 groups (p < 0.001 for both variables).

Both TRAP^+^OcS/BS and TRAP^+^OcN/BS were significantly higher in OVX group versus SHAM, OVX-LR and OVX-HR groups (Fig 2C & 2D). Furthermore, these 2 variables were significantly higher in OVX-LR group than in OVX-HR or SHAM group, but there was no significant difference between the OVX-HR and the SHAM groups (Fig 2C & 2D).

### Bone microarchitecture

**Fig 3:**
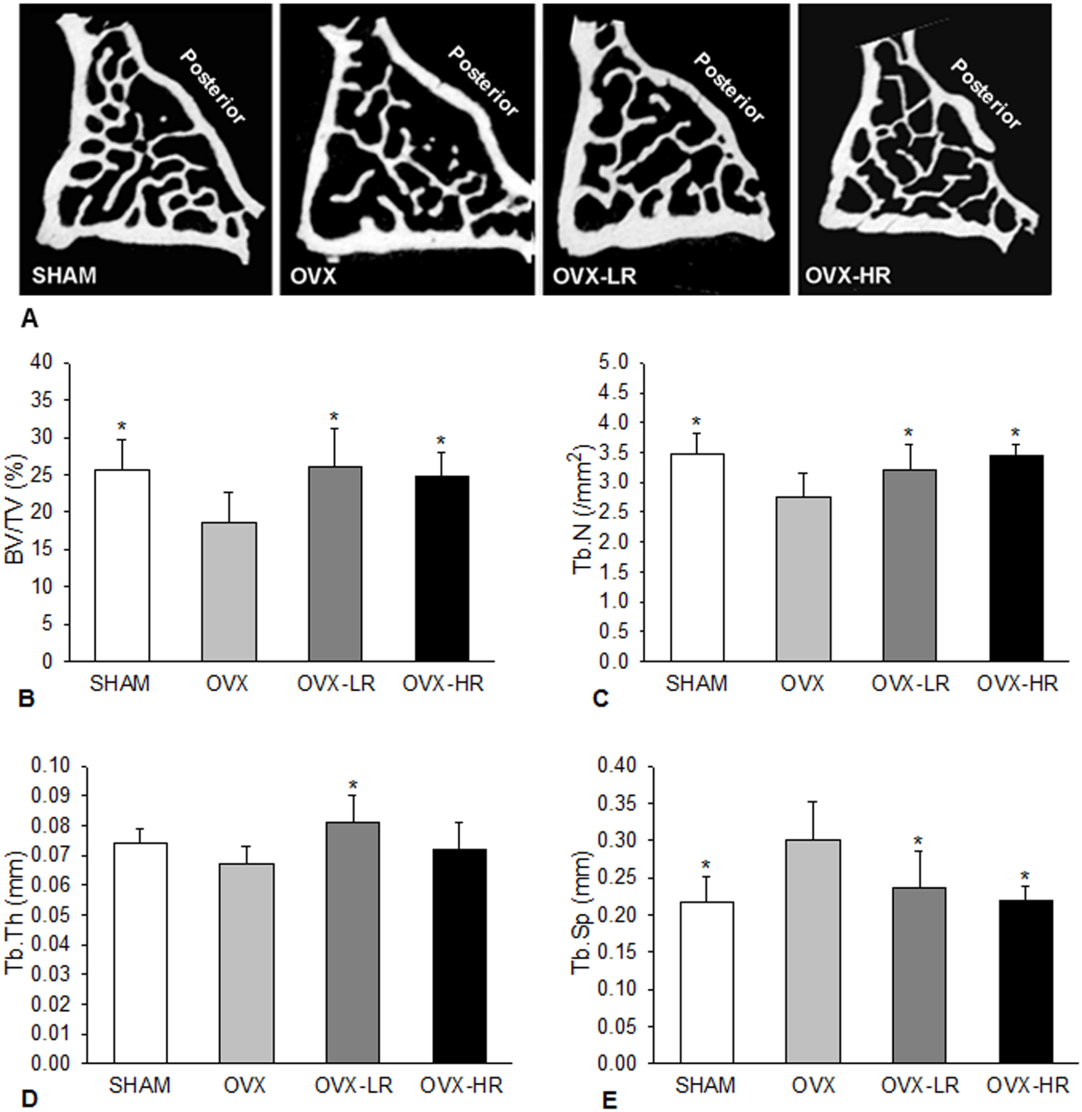
Low-dose RIS preserves cancellous bone structure as good as high-dose RIS in OVX rats. A) 2D micro-CT images of the first lumber vertebra from different groups; B-E) Comparison of bone volume (BV/TV)(*B*), trabecular number (Tb.N)) (*C*), trabecular thickness (Tb.Th)) (*D*) and trabecular separation (Tb.Sp)(*E*), respectively, among SHAM, OVX, OVX-LR and OVX-HR groups. *p < 0.05 *versus* OVX group. Numerical data are presented in supporting information (S3 Table).

The cross-sectional micro-CT images of L1 vertebral bodies from different groups of rats are shown in Fig 3A. As expected, cancellous bone loss occurred in OVX rats treated with vehicle.

Quantitative micro-CT analyses showed that differences in BV/TV, Tb.Th, Tb.N and Tb.Sp in cancellous bone were all significant among the 4 groups (p < 0.01 ~ 0.001)(Fig 3B–3E). Compared to SHAM group, OVX rats showed significant decreases in BV/TV (-27%) and Tb.N (-20%) but significant increase in Tb.Sp (+28%). Both high and low doses of RIS significantly improved cancellous bone structure in OVX rats. There were significant increases in BV/TV and Tb.N and significant decrease in Tb.Sp in OVX rats treated with RIS regardless of the dose used. A significant increase in Tb.Th occurred only in OVX rats treated with low-dose RIS. However, no variable showed significant difference among SHAM, OVX-LR and OVX-HR groups. The micro-CT did not show significant differences in cortical BV/TV and Ct.Th among the 4 groups (numerical data are present in the supporting information- S3 Table).

### Relationship between osteoclast activity and cancellous bone structure

The correlations between osteoclast activity and cancellous bone microarchitecture are shown in Table 1. Both TRAP^+^OcS/BS and TRAP^+^OcN/BS correlated significantly with BV/TV, Tb.N and Tb.SP in pooled samples from SHAM and OVX groups, but not in pooled samples from OVX-LR and OVX-HR groups.

**Table 1:** Correlation between osteoclast- and bone structure-related variables in lumbar vertebral cancellous bone.

		BV/TV	Tb.Th	Tb.N	Tb.Sp
**SHAM+OVX**					
TRAP^+^Oc.S/BS	r	-0.588	-0.475	-0.608	0.610
	p	0.013	0.054	0.010	0.009
TRAP^+^Oc.N/BS	r	-0.605	-0.473	-0.639	0.644
	p	0.010	0.055	0.006	0.005
**OVX-LR+OVX-HR**					
TRAP^+^Oc.S/BS	r	0.140	0.362	-0.241	0.179
	p	0.579	0.140	0.336	0.477
TRAP^+^Oc.N/BS	r	0.058	0.358	-0.378	0.315
	p	0.818	0.145	0.122	0.202

## Discussion

OVX rat is an established animal model for the investigation of postmenopausal osteoporosis and its treatment effects. Our study results demonstrated that both apoptotic osteocytes and empty lacunae in vertebral cancellous bone were significantly increased in OVX rats resulting in a severe decrease in normal osteocytes. In addition, osteoclast number was also significantly increased in OVX rats. Emerton et al [3] reported that osteocyte apoptosis was significantly increased in mice within 3 days after OVX, whereas osteoclastic bone resorption was increased in the regions concomitant with osteocyte apoptosis until 14 days after OVX. This temporal and spatial relationship suggests that increased osteoclastic bone resorption induced by estrogen depletion is linked to osteocyte apoptosis [3]. Apoptosing osteocytes may release ATP as a “find me” signal to stimulate RANKL expression in the neighboring viable osteocytes, which enhances osteoclast number and activity on the bone surface [20, 21]. In general, bone resorption is the primary means of removing dead osteocytes. However, the increase in empty lacunae implies that the rate of bone resorption, although significantly increased after estrogen depletion, may still fall behind the accumulation of apoptotic osteocytes, especially in deep-seated interstitial bone that is less accessible to osteoclasts. The material properties of bone may be compromised with the expansion of accelular areas.

It has been reported that inhibition of osteocyte apoptosis by QVD-OPh, a pan-caspase inhibitor, can significantly suppress OVX-induced bone resorption [3]. Accordingly, we postulate that the use of low-dose BPs after estrogen depletion may decrease bone loss and improve bone quality via inhibition of osteocyte apoptosis. Roelofs et al [22, 23] reported that the fluorescent RIS analogues were found in osteocyte lacunae and canaliculi in rat/mice bone for >7 days after intravenous injection, suggesting that osteocytes were exposed to the administered BP during this period. It is possible that the number of osteocytes coated by BP will increase with repeated dosing and increasing time [22]. Because of its lower affinity, RIS may gain faster access to osteocyte network [24]. Therefore, we selected RIS for the present study. The high-dose RIS was defined by the oral dose used for the treatment of osteoporosis. Since less than 1% of orally administered dose is absorbed [25], the high-dose RIS (0.8 μg/kg/day) injected in rats is approximately equivalent to the oral dose taken by a 60 kg human (5 mg/day or 83.3 μg/kg/day), which can produce optimal bone antiresorptive activity [16]. The low-dose RIS was designated as one tenth of the high dose as previously defined [16]. Our results showed that both low and high-dose RIS can effectively inhibit osteocyte apoptosis and osteoclast activity in OVX rats. However, it is difficult to conclude that low-dose RIS is superior to high-dose RIS in inhibiting OVX-induced osteocyte apoptosis, because we did not find significant differences in apoptotic osteocytes and empty lacunae between OVX rats treated with low- and high-dose RIS. However, only low-dose RIS inhibited osteocyte apoptosis to the level seen in SHAM rats. This suggests that low-dose RIS may provide moderately higher inhibitory effect on osteocyte apoptosis than high-dose RIS. Additionally, our results confirm that high-dose RIS suppresses osteoclast activity much more pronounced than low-dose RIS in OVX rats.

BPs are traditionally viewed as antiresorptive agents that reduce osteoclast activity by inhibiting farnesyl pyrophosphate (FPP) synthase, a key enzyme of the mevalonate pathway [26, 27]. More recently, it has been recognized that BPs can also prevent osteoblast and osteocyte apoptosis by opening Cx43 hemichannels, resulting in activation of the Src/MEK/ERK pathway [26, 28]. *In vitro* studies suggest that such dual effects of BPs are dose-dependent [18]. The clinical doses exclusively inhibit osteoclastogenesis and promote osteoclast apoptosis, but the lower doses that do not affect osteoclasts would play an anti-apoptotic role in osteoblasts and osteocytes [18]. However, the results from this study suggest that the dose-dependent effect of BPs is not so distinct in *in vivo*. In addition to inhibit osteocyte apoptosis, low-dose BPs can also inhibit osteoclast activity in OVX rats. The mechanism by which low-dose BPs suppress osteoclastic bone resorption may be linked to the prevention of osteocyte apoptosis, reducing signals that stimulate RANKL release from neighboring osteocytes [15, 17, 21]. Nevertheless, whether low-dose BPs play direct role in the suppression of osteoclast activity remains unclear.

Compared to the SHAM rats, there were significant changes in BV/TV (-27%), Tb.N (-20%) and Tb.Sp (+28%) in vertebral cancellous bone by 90 days after OVX, which are similar to the results reported by Mathavan et al [29]. Conversely, the cortical thickness and BV/TV were not significantly changed in OVX rats. There was a significantly positive correlation between osteoclast activity and loss of cancellous bone in pooled samples from SHAM and OVX rats, both of which were not treated with RIS. It suggests that bone loss after estrogen depletion is strongly associated with excessive osteoclastic bone resorption. In contrast, this correlation was not significant in pooled samples from OVX-LR and OVX-HR rats, because there is no significant difference in bone volume between these two groups. These results indicate that low-dose BPs preserves cancellous bone volume as good as high-dose BPs, although its effect on osteoclasts is much lower. The reason for low-dose BPs to prevent bone loss is likely to be caused by increased bone formation in the resorption cavities [15], which is supported by the increase in Tb.Th in OVX-LR group. There is evidence that low-dose BPs would stimulate osteoblastogensis and inhibit osteoblast apoptosis to increase bone formation [18, 30, 31]. Additionally, inhibition of osteocyte apoptosis by low-dose BPs may decrease empty lacunae and micropetrosis in bone, resulting in preservation of functional osteocyte network that maintains optimal bone quality [32, 33].

To the best of our knowledge, there is no information regarding the minimal dose of BPs that can protect bone structure and strength in postmenopausal women. The current study indicates that RIS at 1/10 of the clinical dose may prevent bone loss after estrogen depletion. However, inhibition of osteoclast activity by low-dose RIS is much milder than that by high-dose RIS, implying that low-dose BPs may not decrease bone remodeling as deeply as high-dose BPs. The clinical doses of BPs may cause severe suppression of bone remodeling that would compromise bone quality to increase the risk of atypical femoral fracture [34–36]. The low-dose (≈1/10 of clinical dose) regimen of BP treatment, if takes effect in the clinic, is expected to reduce complications resulting from long-term BP therapy on osteoporosis and other diseases.

In conclusion, our study demonstrated that both high and low dose RIS can inhibit osteocyte apoptosis and reduce osteoclast activity in OVX rats. Compared to the high-dose RIS, low-dose RIS has a milder effect on suppression of osteoclast activity. However, low-dose RIS is likely to preserve cancellous bone mass and microarchitecture similar to the high-dose RIS after estrogen depletion.

## Supporting information

S1 Table (DOCX)

S2 Table (DOCX)

S3 Table (DOCX)
