# Supplementary material for: Protective effect of low-dose risedronate against osteocyte apoptosis and bone loss in ovariectomized rats
Source: PLoS One. 2017 Oct 18;12(10):e0186012. doi: 10.1371/journal.pone.0186012 (PMC5646759; doi:10.1371/journal.pone.0186012)
Supplement: S1 Table — (DOCX) [file pone.0186012.s001.docx]

**Supporting information**

S1 Table.

**S1 Table**. Comparison of osteocyte-related variables among 4 groups

|  |  | Casp-3^-.^Ot | Casp-3^+.^Ot | E.Lac |
| --- | --- | --- | --- | --- |
|  |  | % | % | % |
| SHAM | mean | 87.6* | 6.23* | 6.20* |
|  | SD | 2.77 | 1.38 | 1.89 |
|  |  |  |  |  |
| OVX | mean | 76.2 | 12.0 | 11.8 |
|  | SD | 5.49 | 2.59 | 4.23 |
|  |  |  |  |  |
| OVX-LR | mean | 84.8* | 8.31* | 6.85* |
|  | SD | 2.99 | 1.85 | 2.29 |
|  |  |  |  |  |
| OVX-HR | mean | 83.9* | 9.59*^#^ | 6.53* |
|  | SD | 4.20 | 3.51 | 1.33 |
|  |  |  |  |  |
|  | p-value | < 0.001 | < 0.001 | < 0.001 |

Post-Hoc test: *p < 0.05 *versus* OVX group; ^#^p < 0.05 *versus* SHAM group
