# Supplementary material for: Protective effect of low-dose risedronate against osteocyte apoptosis and bone loss in ovariectomized rats
Source: PLoS One. 2017 Oct 18;12(10):e0186012. doi: 10.1371/journal.pone.0186012 (PMC5646759; doi:10.1371/journal.pone.0186012)
Supplement: S2 Table — (DOCX) [file pone.0186012.s002.docx]

**Supporting information**

S2 Table.

**S2 Table.** Comparison of osteoclast-related variables among 4 groups

|  |  | TRAP^+^Oc.S/BS | TRAP^+^Oc.N/BS |
| --- | --- | --- | --- |
|  |  | % | /mm |
| SHAM | mean | 0.830* | 0.187* |
|  | SD | 0.508 | 0.112 |
|  |  |  |  |
| OVX | mean | 7.44 | 1.21 |
|  | SD | 1.28 | 0.147 |
|  |  |  |  |
| OVX-LR | mean | 2.97*^#^ | 0.481*^#^ |
|  | SD | 1.06 | 0.132 |
|  |  |  |  |
| OVX-HR | mean | 0.524*^+^ | 0.076*^+^ |
|  | SD | 0.751 | 0.074 |
|  |  |  |  |
|  | p-value | < 0.001 | < 0.001 |

Post-Hoc test: *p < 0.05 *versus* OVX group; ^#^p < 0.05 *versus*

SHAM group; +p < 0.05 *versus* OVX-LR group
