# Supplementary material for: Protective effect of low-dose risedronate against osteocyte apoptosis and bone loss in ovariectomized rats
Source: PLoS One. 2017 Oct 18;12(10):e0186012. doi: 10.1371/journal.pone.0186012 (PMC5646759; doi:10.1371/journal.pone.0186012)
Supplement: S3 Table — (DOCX) [file pone.0186012.s003.docx]

**Supporting information**

S3 Table.

**S3 Table.** Comparison of bone structure-related variables among 4 groups

|  |  | Cancellous Bone | | | | Cortical Bone | |
| --- | --- | --- | --- | --- | --- | --- | --- |
|  |  | BV/TV | Tb.N | Tb.Th | Tb.Sp | Ct.Th | BV/TV |
|  |  | % | /mm^2^ | mm | mm | mm | % |
| SHAM | mean | 25.7* | 3.46* | 0.074 | 0.218* | 0.164 | 0.807 |
|  | SD | 4.13 | 0.360 | 0.005 | 0.034 | 0.018 | 0.032 |
|  |  |  |  |  |  |  |  |
| OVX | mean | 18.7 | 2.76 | 0.067 | 0.301 | 0.151 | 0.791 |
|  | SD | 3.88 | 0.390 | 0.006 | 0.051 | 0.016 | 0.032 |
|  |  |  |  |  |  |  |  |
| OVX-LR | mean | 25.9* | 3.20* | 0.081* | 0.237* | 0.160 | 0.791 |
|  | SD | 5.34 | 0.433 | 0.009 | 0.049 | 0.018 | 0.046 |
|  |  |  |  |  |  |  |  |
| OVX-HR | mean | 24.7* | 3.45* | 0.072 | 0.219* | 0.147 | 0.768 |
|  | SD | 3.44 | 0.197 | 0.009 | 0.019 | 0.012 | 0.047 |
|  |  |  |  |  |  |  |  |
|  | p-value | 0.001 | 0.003 | <0.001 | <0.001 | NS | NS |

Post-Hoc test: *p < 0.05 *versus* OVX group
